# Deep Eutectic Solvent Synthesis of LiMnPO_4_/C Nanorods as a Cathode Material for Lithium Ion Batteries

**DOI:** 10.3390/ma10020134

**Published:** 2017-02-06

**Authors:** Zhi Wu, Rong-Rong Huang, Hang Yu, Yong-Chun Xie, Xiao-Yan Lv, Jing Su, Yun-Fei Long, Yan-Xuan Wen

**Affiliations:** 1 School of Chemistry and Chemical Engineering, Guangxi University, Nanning 530004, China; wuzhi1219@163.com (Z.W.); 13077702014@163.com (R.-R.H.); liuzhouyuhang@163.com (H.Y.); 15878175845@163.com (Y.-C.X.); sujing928@126.com (J.S.); longyf@gxu.edu.cn (Y.-F.L.); 2 Guangxi Colleges and Universities Key Laboratory of Novel Energy Materials and Related Technology, Nanning 530004, China; 3 The New Rural Development Research Institute, Guangxi University, Nanning 530004, China; lvxiaoyan666@163.com

**Keywords:** lithium ion batteries, cathode materials, LiMnPO_4_, deep eutectic solvents

## Abstract

Olivine-type LiMnPO_4_/C nanorods were successfully synthesized in a chloride/ethylene glycol-based deep eutectic solvent (DES) at 130 °C for 4 h under atmospheric pressure. As-synthesized samples were characterized by X-ray diffraction (XRD), scanning electron microscopy (SEM), transmission electron microscopy (TEM), Raman spectroscopy, Fourier transform infrared spectroscopy (FTIR) and electrochemical tests. The prepared LiMnPO_4_/C nanorods were coated with a thin carbon layer (approximately 3 nm thick) on the surface and had a length of 100–150 nm and a diameter of 40–55 nm. The prepared rod-like LiMnPO_4_/C delivered a discharge capacity of 128 mAh·g^−1^ with a capacity retention ratio of approximately 93% after 100 cycles at 1 C. Even at 5 C, it still had a discharge capacity of 106 mAh·g^−1^, thus exhibiting good rate performance and cycle stability. These results demonstrate that the chloride/ethylene glycol-based deep eutectic solvents (DES) can act as a new crystal-face inhibitor to adjust the oriented growth and morphology of LiMnPO_4_. Furthermore, deep eutectic solvents provide a new approach in which to control the size and morphology of the particles, which has a wide application in the synthesis of electrode materials with special morphology.

## 1. Introduction

Lithium ion batteries (LIBs) have been considered as the most promising power sources for modern electronic devices due to their long cycle life, high energy density and good safety [1,2,3]. The cathode material is an important part of LIBs and plays a critical role in determining their performance [4,5]. Thus, many types of cathode materials have been investigated to improve the performance of LIBs. Since the pioneering work of Goodenough and co-workers [6], LiMPO_4_ (M = Fe, Ni, Mn, Co) with an olivine-type structure has received intensive attention over the past decades [7,8,9]. Among the known olivine-type materials, LiMnPO_4_ has been investigated as a promising cathode material for the next generation of LIBs due to its optimal redox potential (4.1 V vs. Li^+^/Li), giving an energy density that is approximately 20% larger than that of LiFePO_4_ and is compatible with most liquid electrolytes presently used in LIBs [4,6,10,11,12]. However, LiMnPO_4_ suffers from slow lithium ion diffusion (10^−15^ cm·s^−1^) and poor electronic conductivity (10^−10^ cm·s^−1^), due to the heavy polarized holes localized on the Mn^3+^ sites and the interfacial strain that exists between the LiMnPO_4_ and MnPO_4_ phases during charge/discharge processes [13,14,15]. In order to overcome these drawbacks, three approaches have been adopted: (1) reducing the particle size and controlling the morphology [16,17,18]; (2) coating a conductive layer on the surface of LiMnPO_4_ [19,20,21,22]; and (3) doping with cations [23,24,25,26].

According to previous literature [10,27,28,29], nanoparticles and nanostructures are beneficial in increasing capacity, as they can effectively shorten the diffusion length of the Li-ion and electron. Therefore, many approaches have been carried out to prepare LiMnPO_4_/C with nano-size and nanostructure [30,31,32]. In these adopted approaches, hydrothermal/solvothermal methods have attracted particular attention owing to the controllable synthesis of LiMnPO_4_/C with a special morphology [2,20,21,33,34,35]. Therefore, various special morphologies such as nanoplates [16,21,36]; nanorods [10,33,35]; nanosheets [18,37]; flower-like nanostructures [34,38]; hemoglobin [39]; and wedges [40] were prepared using the hydrothermal/solvothermal method to enhance the electrochemical performance of LiMnPO_4_. LiMnPO_4_ nanoplates were synthesized using an ethylene glycol (EG)-assisted solvothermal approach and delivered a discharge capacity of 92 mAh·g^−1^ at 0.5 C [32]. LiMnPO_4_ nanorods were prepared via a facile solvothermal approach, and exhibited a high capacity of 110 mAh·g^−1^ at 10 C [35]. In summary, the use of hydrothermal/solvothermal methods made it easy to control the morphology and size of LiMnPO_4_ particles. However, hydrothermal/solvothermal methods require the cumbersome use of autoclaves and high temperature, which limits their practical application. Recently, the ionothermal method based on ionic liquids (ILs) has been adopted to overcome the drawbacks of the hydrothermal/solvothermal methods, as ILs have low vapor pressure and excellent solvating properties and thus the use of autoclaves can be avoided [41,42,43]. For instance, Barpanda et al. [44] synthesized nanostructure LiMnPO_4_ via an ionothermal route, and the prepared sample exhibited a reversible capacity of 95 mAh·g^−1^ at 1/20 C with good cycling stability. However, the application of the ionothermal method failed to become popular due to limitations such as poor biodegradability, toxicity and high cost [45,46].

**Figure 1:**
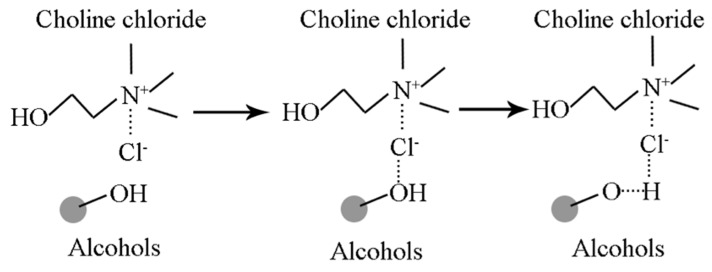
Schematic illustration of the formation mechanism of choline chloride and hydrogen bond in deep eutectic solvents (DESs).

Since the term deep eutectic solvents (DESs) was first coined by Abbott et al. [47,48], they have been investigated as a sustainable media in which to overcome the limitations of ILs [46]. Compared with traditional ILs, DESs can be readily formed by mixing quaternary ammonium or phosphonium salt with a metal salt or hydrogen bond donor (HBD), such as amide, acid or alcohol under simple operational conditions [49,50]. Taking choline chloride/alcohols-based DESs as an example, the formation mechanism of choline chloride and hydrogen bond in DESs is presented in Figure 1 [51].

DESs share most of the same characteristics as ILs, such as negligible vapor pressure, tenability and wide electrochemical potential windows [47,48]. However, DESs have some unique advantages such as nontoxicity and low cost, and they are biodegradable; and easily are prepared [46]. The freezing point of these DESs even can be reduced to a lower temperature than that of ILs due to their low lattice energy [52]. Thus, DESs have widespread applications in the fields of chemistry, including synthesis [46], metal deposition [53,54], nanomaterials [55], gas adsorption [56], analysis [57] and electrochemistry [58]. For example, sulfonamide-based deep eutectic electrolytes display significantly higher transport numbers than organic carbonates electrolyte solutions such as LiPF_6_ [59]. Similarly, Lesch et al. [60] reported that the LiTFSI/urea based deep eutectic electrolyte with high urea concentrations can significantly increase ionic diffusivities and lithium transference numbers. Zinc has been successfully electrodeposited in choline chloride/urea and choline chloride/ethylene glycol deep eutectic solvents, respectively [61], with the morphology of zinc coatings obviously changed in those different choline chloride-based DESs due to their different physical properties. These results indicate that DESs are a potential media that can be used in the synthesis of numerous materials. However, the synthesis of nanostructure LiMnPO_4_ in DESs has not yet been reported. 

In this paper, we prepared LiMnPO_4_/C nanorods in a chloride/ethylene glycol-based deep eutectic solvent (DES) under atmospheric pressure and investigated the electrochemical properties of the prepared sample. The results of this work show that DES can be used as a new structure-directing agent to prepare LiMnPO_4_ with a special rod-like morphology.

## 2. Experimental Methodology

### 2.1. Sample Synthesis

LiMnPO_4_/C nanorods were prepared using H_3_PO_4_ (85% P_2_O_5_), MnSO_4_·H_2_O and LiOH·H_2_O as the raw materials and chloride/ethylene glycol-based DES as the solvent and reaction medium. All chemicals were analytical grade reagents from Sinopharm Chemical Reagent Co. Ltd. (Beijing, China) without further purification. During the synthesis processes, the mole ratio of Li:Mn:P was 3:1:1. The DES was prepared by mixing ethylene glycol and choline chloride with a mole ratio of 2:1 at 80 °C for 30 min to form a colorless liquid [46]. First, a MnSO_4_ saturated solution and H_3_PO_4_ were dissolved in DES. Next, a LiOH saturated solution was added dropwise into the DES (containing MnSO_4_ and H_3_PO_4_) to form a yellowish-brown precursor suspension, where the concentration of Mn^2+^ was controlled at 0.2 mol·L^−1^. The yellowish-brown suspension was then heated at 130 °C for 4 h with the heating rate of 3 °C·min^−1^ under atmospheric pressure to obtain a whitish suspension. After centrifuging the whitish suspension with ethanol and deionized water several times, whitish LiMnPO_4_ (LMP) powders were obtained. In order to improve the conductivity and crystallization of the sample, the collected LMP powders were dispersed in a sucrose solution with a weight ratio of 7.5:2.5 for LMP: sucrose, and the obtained LMP/sucrose suspension was dried with a spray dryer to obtain a dried LMP/sucrose mixture powder. The conditions of the spray drying are as follows: air inlet temperature was 200 °C, air inlet volume was 3 m^3^·min^−1^, feed rate was 1.2 L·h^−1^, the diameter of nozzle was 0.5 mm, and the solid content is 50 g·L^−1^. The LMP/sucrose mixture powder was heated at 300 °C for 1 h and then sintered at 600 °C for 5 h with the heating rate of 5 °C·min^−1^ under N_2_ flow in a tube furnace. Finally, LiMnPO_4_/C (LMP/C) was obtained upon cooling to room temperature.

In order to study the effects of chloride and ethylene glycol, a second LiMnPO_4_/C sample was synthesized using only ethylene glycol as the solvent with other synthetic conditions kept unchanged.

### 2.2. Characterization

X-ray diffraction (XRD) measurements were tested on a X’Pert PRO equipment and used a Cu Kα radiation source (λ = 0.154060 nm) with a scan range of 10° to 80° and a scan step of 0.0065° and 10 wt % standard silicon powder was added to correct the test instrument error. The power morphology was observed using scanning electron microscopy (SEM) in a Hitachi S-4800 instrument (Hitachi, Ltd., Tokyo, Japan) with an accelerating voltage of 3.0 KV and working distance of 4.5 mm; and transmission electron microscope (TEM) in a Tecnai G2 F20 apparatus (FEI Corporation, Hillsboro, OR, USA). The carbon content of the final product was obtained with a Thermo Scientific Flash 2000 elemental analyzer (Thermo Scientific Corporation, Warminster, MA, USA) with a carrier gas velocity of 140 mL·min^−1^; oven temperature of 65 °C; oxygen injection time of 5 s and a running time of 720 s. Raman spectroscopy was undertaken by a Renishaw (Renishaw, London, UK) in Via Reflex with a 785 nm wavelength laser. The Fourier Transform Infrared Spectrum (FT-IR) was tested using a Thermo Nicolet IS50 spectrometer (Thermo Scientific Corporation, Warminster, MA, USA).

### 2.3. Electrochemical Measurements

The prepared LiMnPO_4_/C, acetylene black and a poly (vinylidene fluoride) (PVDF, HSV900, (MTI Corporation, Shenzhen, China) binder sample were mixed with a weight ratio of 80:15:5 in *N*-methyl-2-pyrrolidone (Xilong Chemical Co., Ltd., Guangzhou, China). The slurry was painted onto an aluminum foil current collector with a thickness of 100 μm and dried overnight in a vacuum at 120 °C. The working electrodes were obtained after punching with a diameter of 14 mm, and the surface density of the active material was approximately 1.5 mg·cm^−2^.

The charge/discharge behaviors of the prepared LiMnPO_4_/C were tested using CR2032 coin-type cells assembled in an argon-filled glove box. Lithium foil was used as the counter electrode, Celegard^®^ 2300 as the separator and 1 mol·L^–1^ LiPF_6_/(EC + DEC 1:1 by volume) as the electrolyte. Charge/discharge experiments were carried out on a CT-3008 battery testing system (Neware Technology Limited, Shenzhen, China) at different C rates between 2.5 and 4.5 V (vs. Li/Li^+^) at room temperature (25 °C), where a 1 C rate was 170 mAh·g^−1^. Current densities and specific capacities were calculated based on the mass of the LiMnPO_4_/C of the electrode. A three-electrode cell (Lithium Battery Cell 990-00344, Gamry Instruments, Warminster, PA, USA) was assembled and used for cycle voltammetry (CV) and electrochemical impedance spectroscopy (EIS) measurements. CV tests were performed at scan rates ranging from 0.1–0.4 mV·s^−1^ between 2.5–4.5 V under 25 °C. EIS measurements were carried out using a Gamry PCI4/750 electrochemical workstation (Gamry Instruments, Warminster, PA, USA) with an AC voltage of 5 mV amplitude and a frequency range from 100 kHz to 0.001 Hz. During the CV and EIS experiments, the electrode containing the prepared LiMnPO_4_/C was used as the working electrode and Li was used as both the counter and reference electrode.

## 3. Results and Discussion

### 3.1. Material Identification

**Figure 2:**
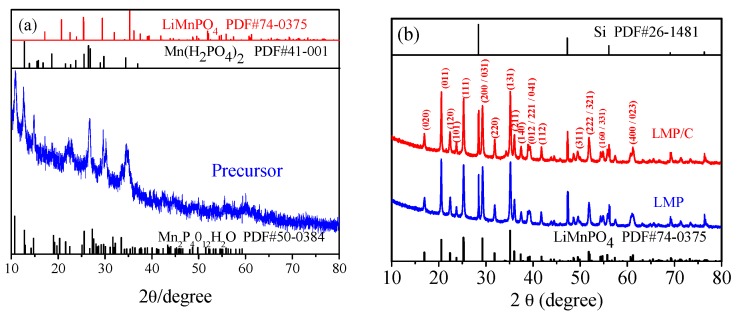
X-ray diffraction (XRD) pattern of (**a**) precursor; and (**b**) LiMnPO_4_ (LMP) and LiMnPO_4_/C (LMP/C).

XRD and FTIR were used to analyze the crystalline structure and chemical composition of the precursor obtained from the yellowish-brown precursor suspension, LMP and LMP/C. The diffraction peaks of the precursor shown in Figure 2a may be ascribed to Mn(H_2_PO_4_)_2_ (PDF# 41-0001) and Mn_2_P_4_O_12_·H_2_O (PDF# 50-0384). It is obvious that a lack of LiMnPO_4_ diffraction peaks and other lithium compounds can be observed in Figure 2a, suggesting that LiMnPO_4_ cannot be formed by mixing the raw materials in DES at room temperature. The XRD patterns of LMP and LMP/C are given in Figure 2b. The Si peak in Figure 2b is in accordance with the standard silicon powder used to correct for instrumentation error during the XRD tests. The peaks shown in Figure 2b can be perfectly indexed to the orthorhombic structure LiMnPO_4_ with a *Pnmb* space group (PDF# 74-0375), indicating that LiMnPO_4_ can be obtained by heating the yellowish-brown precursor suspension at 130 °C for 4 h in DES. The reaction temperature and time used for DES synthesis are much lower than those used in the hydrothermal/solvothermal [27,37,38,39,62] and ionothermal processes [63,64]. The strong and sharp diffraction peaks in Figure 2b demonstrate that both the LiMnPO_4_ and LiMnPO_4_/C samples were well crystallized.

The lattice parameters of LMP and LMP/C are listed in Table 1. The values of the cell volume (*V*) and the lengths (*a*, *b* and *c*) of both samples are close to those in previous studies [65,66]. The values of the lattice parameters of LMP are close to those of LMP/C, indicating that the annealing process has little influence on the lattice parameters. Moreover, the carbon content of LiMnPO_4_/C determined by the CHNS/O elemental analyzer is 9.0 wt %, and no detectable reflections corresponding to carbon are visible in Figure 2b due to its amorphous structure.

**Figure 3:**
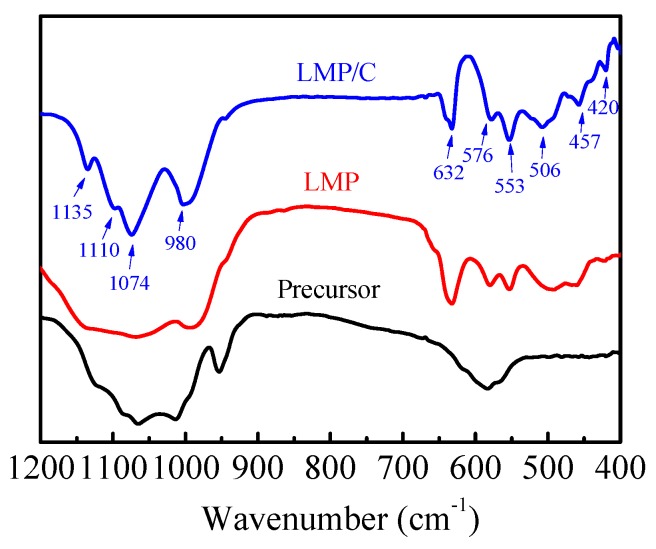
Fourier transform infrared spectroscopy (FTIR) spectra of precursor LMP and LMP/C.

Figure 3 presents the FTIR spectra of the precursor, LMP and LMP/C. According to previous literature [22,67,68], the internal vibrations of LMP/C consisted mainly of four parts [66]: (1) the bands between 1000 cm^−1^ and 1150 cm^−1^,which can be attributed to the anti-symmetric stretching P–O vibrations of the PO_4_^3−^ anion mode (*v*_3_); (2) the band around 980 cm^−1^, which is related to the symmetric PO_4_^3−^ stretching P–O vibration mode (*v*_1_); (3) the bands ranging from 650 cm^−1^ to 550 cm^−1^ can be ascribed to the anti-symmetric bending O–P–O mode (*v*_4_); and (4) the band at 457 cm^−1^ can be associated with the symmetric bending mode (*v*_2_). In addition, the band at 420 cm^−1^ may arise from vibrations of the Mn–O groups [41,67]. The FTIR spectra seen in Figure 3, shows that the precursor contains three type of bands: (1) the band at 576 cm^−1^, which is associated with O–P–O groups; (2) the bands ranging from 980 cm^−1^ to 1135 cm^−1^, which are related to the P–O groups; and (3) the band at 950 cm^−1^ can be ascribed to H–O groups. However, no obvious Li–O characteristic band was observed in the FT-IR spectra of the precursor. The reason for this may be due to the fact that the insoluble solids were MnH_2_P_3_O_16_ and Mn_2_P_4_O_12_·H_2_O and that lithium exists in the form of soluble lithium compounds when the raw materials are mixed in DES at room temperature. After undergoing centrifugation several times, the soluble lithium compounds were left in the solution so that the precursor solid did not contain any lithium compounds. As seen in Figure 3, the LMP and LMP/C have similar FT-IR spectra. However, the intensity of bands of LMP/C was obviously stronger than that of LMP, indicating that the crystallinity of LiMnPO_4_ can be further improved during heat-treatment processes at high temperature [22].

**Figure 4:**
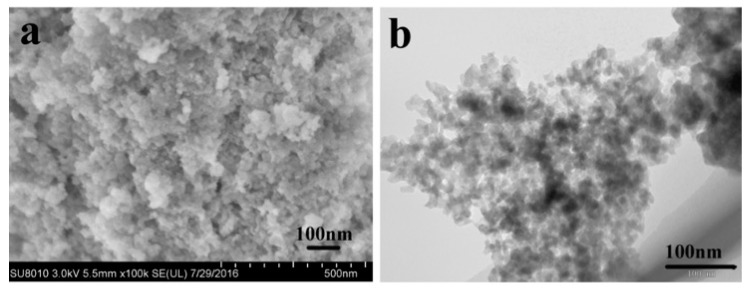

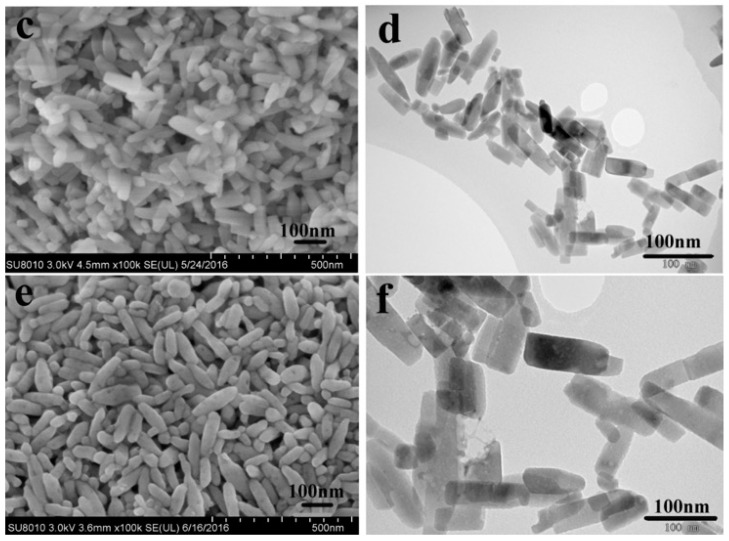
SEM and TEM images of the prepared samples: (**a**,**b**) precursor; (**c**,**d**) LMP; and (**e**,**f**) LMP/C.

SEM and TEM were conducted to identify the change of morphology and size during the synthesis processes. The SEM and TEM images in Figure 4a,b indicate that the precursor powders had no special morphology. From Figure 4c,d, it can be clearly seen that the LiMnPO_4_ particles prepared in DES at 130 °C for 4 h exhibited a rod structure with a length of around 80–110 nm and a diameter of around 30–50 nm, which was similar to the nanorods prepared by the EG-assisted solvothermal approach [27,35,69]. As seen in Figure 4e,f, as-prepared LiMnPO_4_/C maintained the rod-like form (approximately 100–150 nm in length and 40–55 nm in diameter), suggesting that the rod-like structure was sufficiently stable and would not be destroyed during the heat treatment processes [33]. Furthermore, the size of the LiMnPO_4_/C particles increased slightly after annealing, which can be ascribed to the further growth of crystals during the annealing process [35]. As pointed out by Hong et al. [35], the LiMnPO_4_/C nanorods prepared in our work could shorten lithium ions and electron diffusion pathways and provide a large interface between the electrode and electrolyte, which would improve its performance rate.

**Figure 5:**
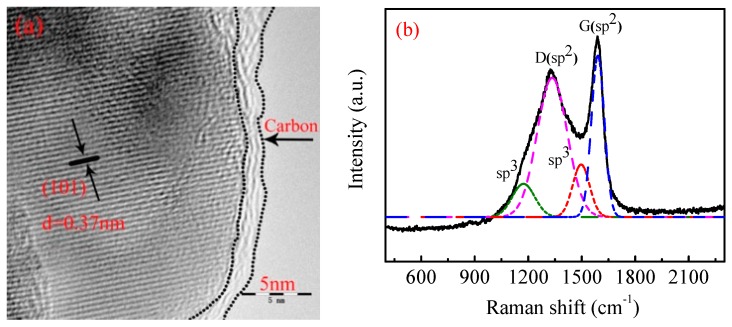
(**a**) HR-TEM image; and (**b**) Raman spectra of the prepared LiMnPO_4_/C.

HR-TEM was used to further observe the crystal structure and the carbon coating layer on the surface of the synthesized LiMnPO_4_/C. As shown in Figure 5a, the clear lattice fringe spacing of 0.37 nm is related to the (101) planes of LiMnPO_4_ (PDF# 74-0375), indicating that the crystallinity of the LiMnPO_4_ was perfect. Additionally, the HR-TEM image also clearly showed that the primary LiMnPO_4_ particles were coated by a thin carbon layer (approximately 3 nm). This thin carbon layer favors the inhibition of the growth of crystal and reduces the agglomeration of the particles [21,33,70], which have been proved by the results in Figure 4e,f. In addition, the LiMnPO_4_/C crystallites were connected directly with the thin carbon layer to form an excellent conducting network, which could effectively enhance the electronic conductivity of LiMnPO_4_/C and further improve its capability rate [12,69].

The structural and physical properties of the carbon layer were further characterized by Raman spectroscopy. The Raman curve of the prepared LiMnPO_4_/C shown in Figure 5b can be fitted with four Gaussian bands located at 1173, 1344, 1496, and 1590 cm^−1^. According to the previous literature [71,72], the band at 1590 cm^−1^ (G band) is the graphitic band and the band at 1344 cm^−1^ (D band) is the disorder band. The existence of the G band and D band indicates a successful coating of carbon on the surface of the LiMnPO_4_/C particles [22]. Both the G band and D band belong to sp^2^-type carbon vibrations, while the other peaks at around 1173 cm^−1^ and 1476 cm^−1^ are associated with sp^3^-type carbon vibrations. The intensity ratio of the G band and D band (*I*_D_/*I*_G_) can be used to characterize the graphitization degree of the carbon materials [22]. In this study, the value of the *I*_D_/*I*_G_ for the prepared LiMnPO_4_/C nanorods was 0.85, which is similar to the value reported by Qin et al. [32] and lower than that reported by Fan et al. [22]. The lower *I*_D_/*I*_G_ value indicates that the carbon layer obtained in our work had a higher degree of graphitization, which is beneficial to electron diffusion and electron conductivity [22].

**Figure 6:**
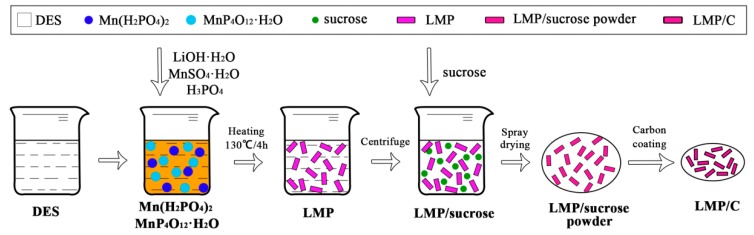
Schematic illustration of the formation of LiMnPO_4_/C nanorods.

The formation processes of the LiMnPO_4_/C nanorods can be schematically illustrated by Figure 6. According to crystal nucleation and growth theory [73], DES plays an important role in the formation of LiMnPO_4_/C nanorods. Initially, PO_4_^3−^ anions react with Mn^2+^ ions to generate insoluble manganese phosphates (mainly Mn_2_P_4_O_12_·H_2_O and Mn(H_2_PO_4_)_2_ particles) when LiOH solution is added to DES at room temperature.

When heated in DES, Li^+^ ions react directly with Mn(H_2_PO_4_)_2_ to form LiMnPO_4_ crystal nuclei, and Li^+^, Mn^2+^, H_2_PO_4_^-^ and OH^-^ react with Mn_2_P_4_O_12_·H_2_O to generate LiMnPO_4_ crystal nuclei. The main reaction equations are described below:

(1) 2LiOH + 2H_3_PO_4_ + MnSO_4_ = Mn(H_2_PO_4_)_2_ + Li_2_SO_4_ + 2H_2_O

(2) 4LiOH + 4H_3_PO_4_ + 2MnSO_4_ = Mn_2_P_4_O_12_ + Li_2_SO_4_ + 8H_2_O

(3) 2LiOH + Mn(H_2_PO_4_)_2_ = LiMnPO_4_ + LiH_2_PO_4_ + 2H_2_O

(4) 10LiOH + Mn_2_P_4_O_12_ + 3MnSO_4_ + LiH_2_PO_4_ = 5LiMnPO_4_ + 3Li_2_SO_4_ + 6H_2_O

During the further crystallization process, we speculated that the DES could adsorb on the surface of the newly formed LiMnPO_4_ as a structure-directing agent to induce the formation of nanorods [50]. In addition, the large viscosity of DES could limit particle size and inhibit the crystal continuous growth by capping the crystal faces during the reaction process [46]. Hence, DES can provide more LiMnPO_4_ nuclei and a lower crystal-oriented growth rate, resulting in the formation of LiMnPO_4_ particles with a rod-like morphology. The specific effects of choline chloride and ethylene glycol on the synthesis of LiMnPO_4_ material will be further discussed in the following section.

### 3.2. Electrochemical Characterization

**Figure 7:**
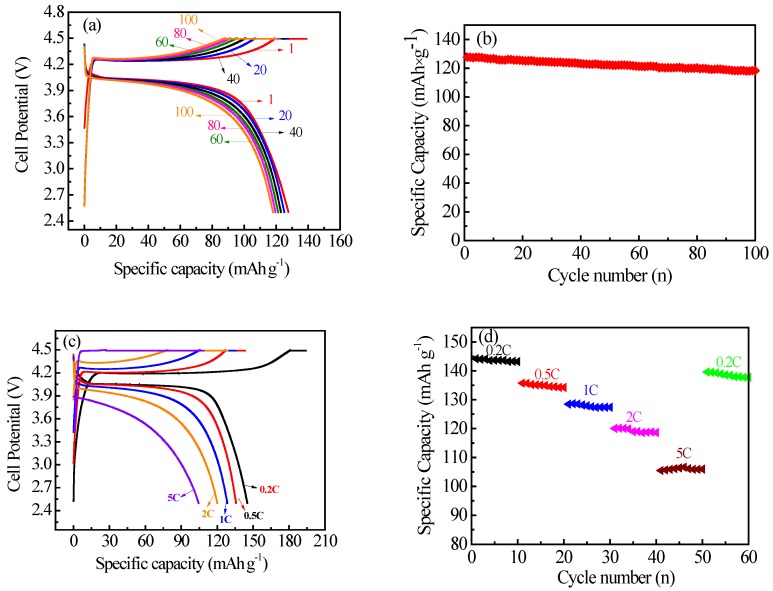
Electrochemical properties for the prepared LiMnPO_4_/C: (**a**) charge-discharge curves at 1 C rate; (**b**) cycle performance at 1 C rate; (**c**) charge-discharge curves at different rates; and (**d**) rate performances at different rates.

Figure 7 shows the charge-discharge behaviors of the prepared LiMnPO_4_/C nanorods. As seen in Figure 7a, the charge-discharge curves of the 1st, 20th, 40th, 60th, 80th and 100th cycles had obvious charge/discharge plateaus around 4.25 V and 4.05 V, which is in accordance with the lithium extraction and insertion processes, respectively [35]. The good overlap of the charge-discharge curves at different cycles shows that the Li^+^ extraction and insertion processes are reversible [35]. The initial charge/discharge specific capacity was 139.0 and 127.9 mAh·g^−1^, respectively, and the initial coulombic efficiency was approximately 92.0%, which is mainly attributed to unavoidable passivation phenomena of the liquid electrolyte at high potential; and side reactions between active materials and electrolytes [16,35,66,74,75]. As seen in Figure 7b, the prepared sample gave an initial discharge capacity of 128 mAh·g^−1^ and maintained over 92.6% of the initial capacity after 100 cycles at 1 C, exhibiting good cycle stability. The improved cycling stability could be due to the nanorod-like morphology, high crystallization and uniform carbon coating.

As shown in Figure 7c, the voltage platform gap between the charge and discharge increased with the increase of the charge-discharge rate, which can be ascribed to the increase of electrode polarization [31]. The charge capacity given in Figure 7c was greater than the theoretical capacity (170 mAh·g^−1^), which can be ascribed to the side reaction of the electrolyte at high potential [16,66]. As seen in Figure 7d, the prepared LiMnPO_4_/C nanorods delivered a discharge capacity of 144, 136, 129 120 and 106 mAh·g^−1^ at 0.2, 0.5, 1, 2 and 5 C, respectively. The rate performance of LiMnPO_4_/C was much better than that of the nanorod-like LiMnPO_4_/C reported in References [76,77], and close to that provided by Hong et al. [35]. The improved performance rate can be attributed to the unique nano-sized rod structure and the carbon layer.

**Figure 8:**
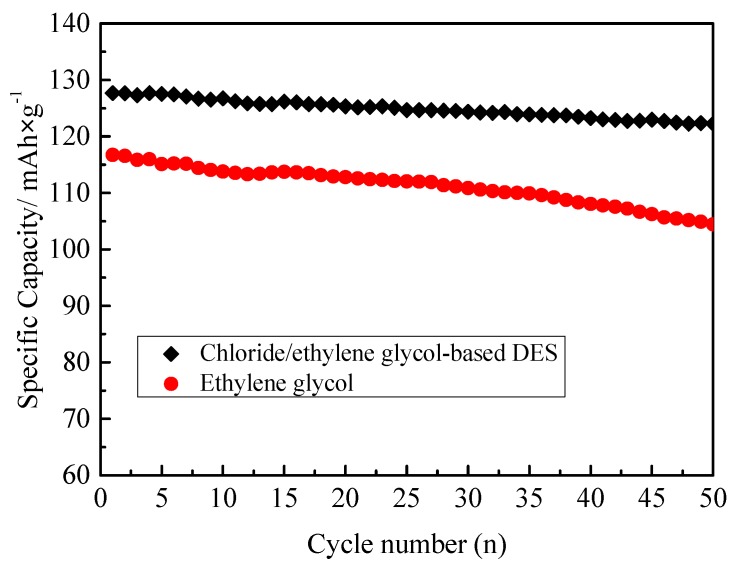
The 1 C rate cycling performance of the LiMnPO_4_/C prepared in DES and EG, respectively.

A comparative experiment was conducted to explore the role of ethylene glycol and choline chloride in the DESs synthesis of LiMnPO_4_. Figure 8 compares the cycling performance of the LiMnPO_4_/C prepared in ethylene glycol/choline chloride and ethylene glycol solvent, respectively. As shown in Figure 8, the LiMnPO_4_/C prepared in ethylene glycol solvent gave a specific discharge capacity of 117 mAh·g^−1^ with a capacity retention ratio of 88% after 50 cycles at 1 C, which is close to that of LiMnPO_4_/C prepared in the ethylene glycol solvent [18,30,33], while the LiMnPO_4_/C prepared in ethylene glycol/choline chloride delivered 128 mAh·g^−1^ with a capacity retention ratio of 95% after 50 cycles at 1 C. The performance of LiMnPO_4_/C prepared in ethylene glycol/choline chloride was much better than that in ethylene glycol, indicating that choline chloride plays a more important role during the synthesis of LiMnPO_4_ in the DES. However, the interaction of choline chloride and ethylene glycol in the DES is under further investigation.

**Figure 9:**
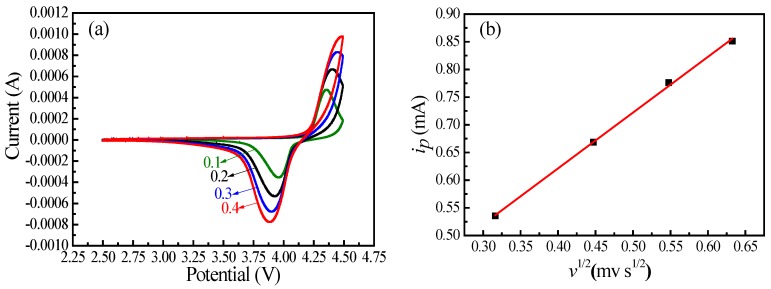
(**a**) Cycle voltammetry curve of the prepared LiMnPO_4_/C; and (**b**) the plots of peak current density (*i*_p_) as a function of the square root of the scan rate (*v*^1/2^).

CV and EIS were carried out to further understand the electrode reaction of the prepared LiMnPO_4_/C. Figure 9 shows the CV behaviors with a scan rates range from 0.1 to 0.4 mV·s^−1^. As seen in Figure 9a, the CV curves present obvious redox peaks corresponding to the extraction and insertion of lithium ions during the charge/discharge processes [33,35]. When the scan rate increased, the cathodic peak moved to the low potential direction and the anodic peak moved to the high potential direction due to the increase in electrochemical polarization [35,70]. The plot of the anodic peak current densities (*i*_p_) compared with the square root of the scan rates (*v*^1/2^) is presented in Figure 9b.

The correlation coefficient (*R*^2^ = 0.999) indicates the relationship between *i*_p_ and *v*^1/2^ is consistent with the Randles-Serick equation [78,79]. The Randles-Sevcik equation can be expressed as the following [78,79]:

(5) $$i_{p}=2.69\times 10^{5}n^{3/2}C_{\mathrm{Li}}AD^{1/2}v^{1/2}$$

where *i*_p_ is the peak current density (A·g^−1^); *n* is the number of electrons involved in the redox process (*n* = 1 for Mn^2+^/Mn^3+^ redox pair); *C*_Li_ is the initial concentration of lithium ions in LiMnPO_4_ (0.022 mol·cm^−3^); *A* is the total surface area of the electrode active material (cm^2^); *D* is the lithium ion diffusion coefficient (cm^2^·s^−1^); and *v* is the scan rate. The value of the lithium ion diffusion coefficients can be calculated from the slope of Equation (5) and the value of the *D* for the prepared LiMnPO_4_/C nanorods was 8.61 × 10^−15^ cm^2^·s^−1^.

**Figure 10:**
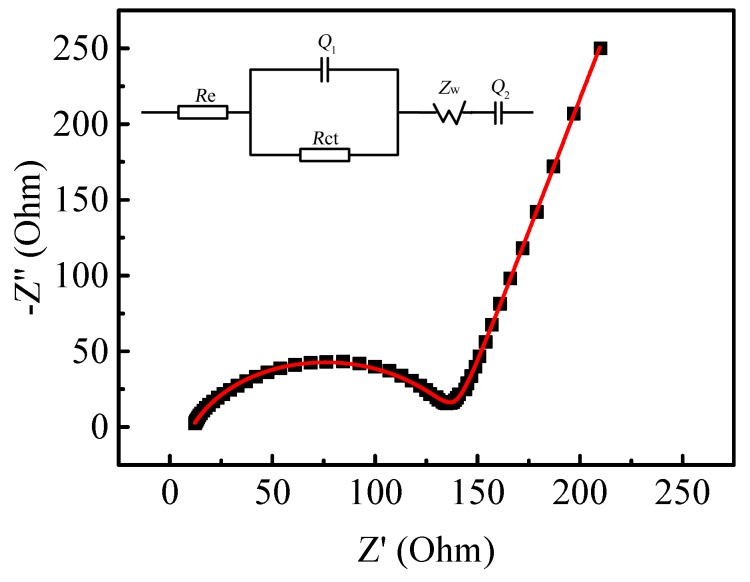
Electrochemical impedance spectra of the prepared LiMnPO_4_/C.

The Nyquist plot of the prepared LiMnPO_4_/C and the corresponding equivalent circuit are shown in Figure 10. The Nyquist curve consists of a compressed semicircle in the high-frequency region related to charge transfer impedance and a declined line in the low-frequency region ascribed to the Li^+^ diffusion resistance (Warburg impedance) in the LiMnPO_4_/C material [30]. The equivalent circuit of EIS is shown in Figure 10, where *R*_e_ stands for the resistance of the electrolyte; *R*_ct_ and *Q*_1_ (Constant phase angle element) are the charge transfer impedance and its electric capacitance of the electrode interface, respectively; *Z*_w_ is known as the Warburg impedance; and *Q*_2_ (constant phase angle element) represents the “dispersion effect” resulting from the Li^+^ diffusion [69,80,81,82]. The values of the parameters of the equivalent circuit are presented in Table 2. As seen in Table 2, *R*_e_ (10.93 Ω) and *R*_ct_ (128 Ω) of the prepared LiMnPO_4_/C nanorods are close to that reported by Qin et al. [32]. The smaller values of *R*_e_ and *R*_ct_ indicate that the nanorods coated with a thin carbon layer can facilitate electrolyte transport and the charge transfer reaction.

The diffusion coefficient of the lithium ions (*D*_Li_) can be calculated according to the following equation [66,75]:

(6) $$D_{\mathrm{Li}}=\frac{R^{2}T^{2}}{2A^{2}n^{4}F^{4}C^{4}\sigma^{2}}$$

where *R* is the gas constant; *T* is the thermodynamic temperature used in the test; *A* is the reaction area of the electrode; *n* is the number of transferred electrons per LiMnPO_4_ molecule during oxidization; *F* is the Faraday constant; *C* is the concentration of lithium ions; and σ represents the Warburg factor, which is the slope of the line between *Z*’ and ω^−1/2^. The value of the *D*_Li_ for the prepared LiMnPO_4_/C was calculated to be 8.91 × 10^−15^ cm^2^·s^−1^, which is larger than that of the LiMnPO_4_/C nanorods synthesized via a facile EG-assisted solvothermal approach [35] and is closed to the *D* value with CV test (8.61 × 10^−15^ cm^2^·s^−1^). The low charge transfer resistance and high diffusion coefficient further prove that as-prepared LiMnPO_4_/C nanorods can display good electrochemical performance.

## 4. Conclusions

In this work, we have successfully prepared olivine-type LiMnPO_4_/C nanorods with an orthorhombic structure in chloride/ethylene glycol-based DES at 130 °C for 4 h. The prepared LiMnPO_4_/C nanorods delivered a discharge capacity of 128 mAh·g^−1^ with a capacity retention ratio of approximately 93% after 100 cycles at 1 C. Even at 5 C, the sample still provided a discharge capacity of 106 mAh·g^−1^, exhibiting a good capability rate and cycling stability. The improved electrochemical performance can be attributed to the rod-like nanostructure and a thin carbon layer coated on the surface of LiMnPO_4_. The results provided in this work demonstrate that chloride/ethylene glycol-based DES can act as a novel structure-directing agent to influence crystal growth orientation and control the micromorphology of LiMnPO_4_. Furthermore, DESs have potential application in the preparation of olivine-type LiMPO_4_ and other electrode materials with a special micromorphology for LIBs.

## Figures and Tables

**Table 1:** Lattice parameters of the prepared LMP and LMP/C.

Sample	*a* (Å)	*b* (Å)	*c* (Å)	*V* (Å^3^)
LMP	10.4437	6.0980	4.7424	302.0
LMP/C	10.4439	6.1021	4.7430	302.3

**Table 2:** Parameters of EIS spectroscopy of the prepared LiMnPO_4_/C.

Element	*R*_e_ (Ω)	*R*_ct_ (Ω)	*Q*_1_ (F)	*n*_1_	*Q*_2_ (F)	*n*_2_
Values	10.93	128.00	1.55 × 10^−6^	0.8	2.18 × 10^-3^	0.8
Error (%)	0.93	1.13	4.15	0.59	4.76	2.67
